# “Other risks don't stop”: adapting a youth sexual and reproductive health intervention in Zimbabwe during COVID-19

**DOI:** 10.1080/26410397.2022.2029338

**Published:** 2022-02-22

**Authors:** Constance R. S. Mackworth-Young, Constancia Mavodza, Rangarirayi Nyamwanza, Maureen Tshuma, Portia Nzombe, Chido Dziva Chikwari, Mandikudza Tembo, Ethel Dauya, Tsitsi Apollo, Rashida A. Ferrand, Sarah Bernays

**Affiliations:** a Assistant Professor, Biomedical Research and Training Institute, Harare, Zimbabwe; Assistant Professor, Department of Global Health and Development, Faculty of Public Health and Policy, London School of Hygiene and Tropical Medicine, London, UK; b Research Fellow, Biomedical Research and Training Institute, Harare, Zimbabwe; PhD Candidate, Department of Global Health and Development, Faculty of Public Health and Policy, London School of Hygiene and Tropical Medicine, London, UK; c Research Assistant, Biomedical Research and Training Institute, Harare, Zimbabwe; d Study Coordinator, Biomedical Research and Training Institute, Harare, Zimbabwe; Assistant Professor, Department of Clinical Research, Faculty of Infectious and Tropical Diseases, London School of Hygiene and Tropical Medicine, London, UK; e Research Fellow, Biomedical Research and Training Institute, Harare, Zimbabwe; PhD Candidate, MRC Tropical Epidemiology Group, Faculty of Epidemiology and Population Health, London School of Hygiene and Tropical Medicine, London, UK; f Study Coordinator, Biomedical Research and Training Institute, Harare, Zimbabwe; g Deputy Director, AIDS and TB Unit, Ministry of Health and Child Care, Central Avenue, Harare, Zimbabwe; h Professor - International Health, Biomedical Research and Training Institute, Harare, Zimbabwe; Professor - International Health, Department of Clinical Research, Faculty of Infectious and Tropical Diseases, London School of Hygiene and Tropical Medicine, London, UK; i Associate Professor, Department of Global Health and Development, Faculty of Public Health and Policy, London School of Hygiene and Tropical Medicine, London, UK; Senior Lecturer in Global Health, School of Public Health, University of Sydney, Australia. *Correspondence*: sarah.bernays@sydney.edu.au

**Keywords:** youth, young people, sexual and reproductive health, HIV, COVID-19, youth-friendly, Zimbabwe

## Abstract

COVID-19 threatens hard-won gains in sexual and reproductive health (SRH) through compromising the ability of services to meet needs. Youth are particularly threatened due to existing barriers to their access to services. CHIEDZA is a community-based integrated SRH intervention for youth being trialled in Zimbabwe. CHIEDZA closed in March 2020, in response to national lockdown, and reopened in May 2020, categorised as an essential service. We aimed to understand the impact of CHIEDZA’s closure and its reopening, with adaptations to reduce COVID-19 transmission, on provider and youth experiences. Qualitative methods included interviews with service providers (*n* = 22) and youth (*n* = 26), and observations of CHIEDZA sites (*n* = 10) and intervention team meetings (*n* = 7). Analysis was iterative and inductive. The sudden closure of CHIEDZA impeded youth access to SRH services. The reopening of CHIEDZA was welcomed, but the necessary adaptations impacted the intervention and engagement with it. Adaptations restricted time with healthcare providers, heightening the tension between numbers of youths accessing the service and quality of service provision. The removal of social activities, which had particularly appealed to young men, impacted youth engagement and access to services, particularly for males. This paper demonstrates how a community-based youth-centred SRH intervention has been affected by and adapted to COVID-19. We demonstrate how critical ongoing service provision is, but how adaptations negatively impact service provision and youth engagement. The impact of adaptations additionally emphasises how time with non-judgemental providers, social activities, and integrated services are core components of youth-friendly services, not added extras.

## Résumé

Le COVID-19 menace les progrès durement acquis dans la santé sexuelle et reproductive (SSR) car il compromet la capacité des services de répondre aux besoins. Les jeunes sont particulièrement à risque, du fait des obstacles contrariant leur accès aux services. CHIEDZA est une intervention communautaire intégrée de SSR pour les jeunes qui est en cours d’expérimentation au Zimbabwe. CHIEDZA a fermé en mars 2020, suite au confinement national, et a rouvert en mai 2020, en tant que service essentiel. Nous souhaitions comprendre l’impact de la fermeture de CHIEDZA et de sa réouverture, avec des adaptations pour réduire la transmission du COVID-19, sur les expériences des prestataires et des jeunes. Les méthodes qualitatives comprenaient des entretiens avec des prestataires de services (*n* = 22) et des jeunes (*n* = 26), ainsi que des observations des sites de CHIEDZA (*n* = 10) et des réunions avec les équipes d’intervention (*n* = 7). L’analyse a été itérative et inductive. La brusque fermeture de CHIEDZA a empêché les jeunes d’avoir accès aux services de SSR. La réouverture de CHIEDZA a été saluée, mais les adaptations requises ont eu des répercussions sur l’intervention et la participation au projet. Les adaptations ont restreint le temps passé avec les prestataires de soins de santé, ont accentué la tension entre le nombre de jeunes ayant accès aux services et la qualité des services. La suppression des activités sociales, qui plaisaient particulièrement aux jeunes hommes, a sapé la participation des jeunes et l’accès aux services, spécialement des garçons. Cet article décrit comment une intervention de SSR communautaire axée sur les jeunes a été touchée par le COVID-19 et s’est adaptée. Nous démontrons combien est importante la continuité de la prestation des services, mais comment les adaptations nuisent à la prestation des services et à la participation des jeunes. L’impact des adaptations souligne également comment les moments passés avec des prestataires non moralisateurs, les activités sociales et les services intégrés sont des éléments clés de services adaptés aux jeunes, et non de simples suppléments.

## Resumen

COVID-19 pone en peligro los avances obtenidos con tanto esfuerzo en el área de salud sexual y reproductiva (SSR) al comprometer la capacidad de los servicios para atender las necesidades de las personas. La juventud en particular se ve amenazada debido a las barreras que enfrenta para acceder a los servicios. CHIEDZA es una intervención de SSR integrada y comunitaria para jóvenes que está siendo puesta a prueba en Zimbabue. CHIEDZA cerró en marzo de 2020, en respuesta al cierre nacional, y volvió a abrir en mayo de 2020, categorizada como un servicio esencial. Nos propusimos entender el impacto del cierre de CHIEDZA y su reapertura, con adaptaciones para reducir la transmisión de COVID-19, en las experiencias de prestadores de servicios y jóvenes. Los métodos cualitativos utilizados fueron entrevistas con prestadores de servicios (*n* = 22) y jóvenes (*n* = 26), y observaciones de los sitios de CHIEDZA (*n* = 10) y reuniones del equipo de intervención (*n* = 7). El análisis fue iterativo e inductivo. El cierre repentino de CHIEDZA impidió el acceso de la juventud a los servicios de SSR. La reapertura de CHIEDZA fue bienvenida, pero las adaptaciones necesarias impactaron la intervención y la participación en ella. Las adaptaciones restringieron el tiempo con los prestadores de servicios de salud, lo cual aumentó la tensión entre la cantidad de jóvenes que accedían al servicio y la calidad de la prestación de servicios. La eliminación de actividades sociales, que habían atraído a los hombres jóvenes en particular, impactó la participación de la juventud y su acceso a los servicios, en particular para los hombres. Este artículo demuestra cómo una intervención de SSR comunitaria, centrada en jóvenes, ha sido afectada y adaptada por COVID-19. Demostramos que la continuación de la prestación de servicios es imperativa, pero que las adaptaciones impactan negativamente la prestación de servicios y la participación de jóvenes. El impacto de las adaptaciones también hace hincapié en que el tiempo con prestadores sin prejuicios, las actividades sociales y los servicios integrados son componentes fundamentales de los servicios amigables a jóvenes, y no extras innecesarios.

## Introduction

With the advent of the global COVID-19 pandemic, the public health measures to reduce transmission, alongside the strain on already under-resourced healthcare systems, are impacting sexual and reproductive health (SRH), including HIV, services.^1–3^ This impact is likely to derail gains made in reducing HIV-related morbidity and mortality^4,5^ and reduce access to family planning services and products.^2^ Sexual and reproductive health (SRH) needs continue and may increase during the COVID-19 pandemic, for example, due to rises in intimate partner violence.^6,7^

The restricted access to SRH services due to COVID-19 particularly threatens youth, who already faced extra barriers in accessing SRH services. The most recent Demographic and Health Survey data from Zimbabwe show that 51% of 15–19 year-olds and 19% of 20–24 year-olds have an unmet need for family planning, 32% of 15–19-year-olds and 7.3% of 20–24-year-olds are living with HIV, and 22% of women aged 15–19 years have begun childbearing.^8^ Such unmet needs are critical, given that youth has been acknowledged as a key stage for developing healthy SRH behaviours.^9^ There has been some progress in addressing the social and structural conditions which impede youth access to SRH services.^10–12^ Many programmes are attempting to adapt to continue service provision to minimise the threat COVID-19 poses,^13^ but the question remains of how we can provide adapted services without abandoning our learning on what works for service delivery for youth.

CHIEDZA is a community-based intervention to improve HIV outcomes in youth (aged 16–24 years), currently being evaluated through a cluster randomised trial in Zimbabwe.^14^ CHIEDZA offers integrated SRH and HIV services, including HIV testing, linkage to treatment, adherence support, as well as family planning, menstrual hygiene management, STI screening and treatment, condoms, pregnancy testing, relationship advice, and counselling. The CHIEDZA intervention closed when the Zimbabwe government mandated a national lockdown on 30th March 2020. The intervention reopened, as an essential service, on 18th May 2020, and has continued to adapt to the changing landscape of COVID-19 and national restrictions.

In this paper, we discuss how CHIEDZA is adapting in the context of COVID-19. We present findings from an ongoing process evaluation and discuss the implications for the delivery of accessible and acceptable services.

## Methods

### Study context

**Figure 1.**
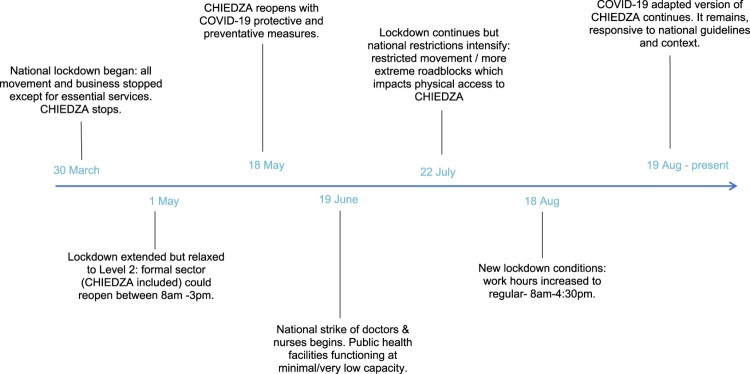
Timeline of Zimbabwe’s national COVID-19 response and CHIEDZA intervention changes (March–August 2020)

When the national lockdown began in Zimbabwe, CHIEDZA stopped all services (Figure 1). On 1st May, the lockdown was extended but relaxed to level 2, allowing specific businesses and additional service providers deemed as essential to reopen. An adapted version of CHIEDZA, in compliance with national guidelines, reopened on the 18th of May, after authorities recognised it as an essential service. As CHIEDZA primarily delivers physical commodities (for example condoms, HIV tests and treatment, and menstrual hygiene products) to young people, beyond SRH information and support, virtual adaptations were not considered feasible, and the focus was on reopening physical service provision in the immediate response to the COVID-19 pandemic. However, some information was subsequently provided using a mobile-based information text service, coinciding with the need that arose because of COVID-19. In June, a national strike of government doctors and nurses began, resulting in public health facilities functioning at minimal or low capacity, including in CHIEDZA communities. This rapidly shifting environment has meant that CHIEDZA has had to be responsive and adaptive throughout the study period.

### CHIEDZA intervention

CHIEDZA is a community-based integrated SRH and HIV intervention for youth (aged 16–24 years) in three provinces in Zimbabwe: Harare, Mashonaland East, and Bulawayo. The aim of CHIEDZA is to improve population-level HIV viral load suppression. The intervention rests on three pillars:

1. Access: community-based youth-friendly settings;
2. Uptake and acceptability: service branding, confidentiality, and social activities;
3. Content and quality: integrated HIV care cascade, high-quality products, and trained providers.

The intervention is delivered in four sites within each of the three provinces (total of 12 sites) by a team of trained healthcare providers, consisting of two nurses, four community health workers, two youth workers, and one counsellor, who work within each site. Youth community mobilisers were recruited from among those regularly using CHIEDZA to encourage other youth from within their communities to attend CHIEDZA. The implementation of the intervention began in 2019 and the implementation and study team hold monthly de-brief team meetings.

When CHIEDZA reopened in May 2020, infection prevention control measures were instigated which altered some aspects of the intervention. All services were moved from inside the community centres to outside, health booth tents were spaced apart and had one wall open for ventilation. To preserve confidentiality, where feasible, the open wall faced a building or was positioned not to be visible to persons outside the tents. Masks were worn by all providers and clients, table surfaces were wiped after use, and handwashing facilities were offered. Social distancing rules were applied, with clients waiting 1–2 m apart, a maximum of three clients registered at a time, and clients being required to leave the CHIEDZA site immediately after they had received services, rather than socialising afterwards with friends as they had done previously. The hours of service provision were changed from 11am to 6pm, which youth had stipulated to fit with their schedules, to 9 am to 3 pm, in order to comply with government-mandated working hours. Additionally, social activities, including pool, darts, football matches and fashion shows, were stopped to enable physical distancing. The package of health services offered remained the same.

### Study design

As part of the trial, a process evaluation has sought to understand implementation, uptake of services, context, and mechanisms of impact. At the beginning of the COVID-19 pandemic, the process evaluation of the trial rapidly and flexibly adapted methods to capture data on the impact of COVID-19 on the intervention and on the experience of youth clients. For this, data collection was iterative, and preliminary analysis of each phase of data collection fed into subsequent phases. We outline the events during the particular time period of data collection in Figure 1, cognisant of the historical specificity and local context which influenced the intervention and provider and youth’s experiences of the CHIEDZA intervention.

### Data collection

Data collection consisted of four parts: (1) interviews with providers, (2) interviews with clients (including community mobilisers), (3) non-participant observation of CHIEDZA sites, and (4) participant observation of regular CHIEDZA study team meetings (Table 1). Interviews were conducted by three trained qualitative researchers (CM, RN, PN), who were part of the process evaluation research team, but separate from the CHIEDZA provider team. Written consent was obtained for the interviews, and verbal confirmation was obtained for the non-participant observations. For the latter, providers were informed of the dates and times when researchers would be present for observations.

**Table 1:** . Data collection methods

Part of data collection	Method	Participants	Number	Time period of data collection	Format of data collection	Status of CHIEDZA intervention	Status of national response to COVID-19
1	Interviews	Healthcare providers	17	March–April 2020	By phone	During the period when CHIEDZA was closed	National lockdown
	Interviews	Healthcare providers	15	July–August 2020	In person (*n* = 10) and by phone (*n* = 5)	2–3 months after CHIEDZA reopened	Tightening of restrictions again; healthcare worker strikes nationally
2	Interviews	Clients	36	May–June 2020	In person	Within the first month of CHIEDZA reopening	Essential services only opened
3	Non-participant observation of CHIEDZA sites	People at CHIEDZA sites include clients and providers	10	May–June 2020	In person	Within the first month of CHIEDZA reopening	Essential services only opened
4	Participant observation of de-brief meetings	CHIEDZA study team and providers	7	March–September 2020	In person and meeting notes	From the reopening of CHIEDZA and during subsequent iterative adaptation	Fluidity and change throughout the period of data collection

#### Interviews with providers

Interviews were conducted with the CHIEDZA healthcare providers at two time points. Firstly, 16 interviews were conducted during the week after the CHIEDZA trial temporarily closed, which was the first week that the government-mandated lockdown started (week beginning 30th March 2020). Six males and ten females were interviewed, with an age range of 24–54 years, and median age of 30.5 years. These interviews sought to understand providers’ perspectives on COVID-19 and the government-mandated lockdown, and the impact of the trial closure on them personally. Fifteen healthcare providers were interviewed two months after CHIEDZA had reopened (July 2020). Five males and ten females were interviewed, with an age range of 24–55 years, and median age of 30 years. These later interviews aimed to understand the providers’ experiences of reopening CHIEDZA, including their perceptions of how the intervention had changed in response to COVID-19-related restrictions and any impact this had. During the lockdown (phase 1), all interviews were conducted by phone. In the second phase of interviews, some interviews were conducted by phone, others in person, depending on the national social distancing and travel restrictions in place at the date of the interview. As the group of healthcare providers from which participants were recruited is small, details of age and gender are not reported, to protect their anonymity.

#### Interviews with clients and community mobilisers

After CHIEDZA had reopened, interviews were conducted with youth clients (*n* = 26). A total of 13 males and 13 females were interviewed, with an age range of 17–24 years and median age of 21 years. Interviews aimed to understand clients’ experience of the adapted CHIEDZA intervention, and how these influenced their interactions with the intervention. Interviews were conducted in person, outdoors at the CHIEDZA sites, where the mobilisers were already working with CHIEDZA, with masks worn.

#### Non-participant observation of CHIEDZA sites

In the first month of reopening (from 18th May 2020), non-participant observations were conducted at CHIEDZA sites across the three provinces. These focused on the adaptations to CHIEDZA that had been made in the context of the COVID-19 pandemic. Non-participant observations were conducted in person, with social distancing and mask-wearing. Non-participant observation note-taking was guided by a template form which detailed the setting, changes related to COVID-19, the process of services at CHIEDZA, interactions between healthcare providers and clients, and general reflections.

#### Participant observation of study team meetings

Participant observations of the research and implementation study team meetings were conducted. These aimed to understand the internal processes that led to adaptations of the intervention, as well as understanding the study team and provider perceptions of the intervention as it adapted. These meetings were also used as an opportunity to feed back some preliminary findings from the process evaluation to the study team, to influence decisions on adaptations to service delivery going forward. Study team meetings were recording through note-taking. Study team meetings were conducted and observed either in person, with social distancing and mask-wearing, or virtually, depending on national restrictions on meetings at the time. For all methods, while mask-wearing and virtual methods impacted understanding facial expressions, and increased reliance on audible cues, they did facilitate data collection during the COVID-19 pandemic.^15^

### Data analysis

Interviews were conducted in the language participants chose and felt most comfortable conversing in (English, Shona or Ndebele). These were audio recorded and transcribed into English by experienced transcribers. Observations were captured through detailed notes. Analysis was driven by a combined thematic and inductive approach,^15^ with pre-determined analytical questioning on how COVID-19 impacted CHIEDZA, and inductive themes emerging within this. Top-level themes included deductive themes: impact of CHIEDZA closure, infection prevention and control adaptations, and context of COVID-19 restrictions; as well as inductive themes: gendered impact of adaptations, mobilisation to attract clients, and retention of clients for repeat visits. CMY led the analysis, and themes emerging from the transcripts and observation notes were discussed in weekly analysis meetings with the process evaluation team (CM, RN, MT, PN, and SB). Continuous attention was paid in these discussions to the researchers’ role in shaping the data collected and the relationships between them and the providers and clients. The research team was not part of the intervention team, which enabled analysis separate from the intervention. For the interviews with the healthcare providers, the researcher who has the closest relationship with the healthcare providers was selected to conduct the interviews with this group, particularly for those interviews conducted virtually, where it is hardest to build rapport without existing relationships.

Themes which emerged from the process evaluation team discussions were iteratively included as a focus for further exploration in subsequent data collection. Data from each source were initially manually coded (by CMY) and grouped according to the themes developed through the coding process and team analytical discussions. This was used to develop a coding framework, with key themes and subthemes. The full dataset of transcripts and observation notes was then coded by CMY using this coding framework in Nvivo12. To support qualitative rigour, we triangulated from multiple data sources, and ideas throughout the analysis process were captured using analytical memos, which were shared across the research team to transparently convey the analysis process and development of key findings.

### Ethical approval

Ethical approval was granted by Medical Research Council of Zimbabwe (MRC/A/2266, 5 November 2018), London School of Hygiene and Tropical Medicine (14652, 21 February 2019) and Biomedical Research and Training Institute (AP144/2018, 23 November 2018). All participants provided written informed consent.

## Results

Quotes under each theme are presented in Table 2.

**Table 2:** . Supporting quotes by results theme

Themes	Quotes
**Disrupted access to SRH services for youth**	
Reduced access to SRH services	“*The condoms that I had actually finished. And I feared going to the clinic because of what my friends said about how the nurses treated them plus since we were on lockdown, I feared the police as we were not allowed to move around*.” (Interview with client, male, 19 years) “*Some boys in my neighbourhood were actually engaging in unprotected sex*” (Interview with client, female, 24 years) “*So, when CHIEDZA was closed, there wasn’t any free supply of family planning and I think there are some people who gave birth and fell pregnant to COVID children. There weren’t any people to hand out family planning pills or condoms so I’m sure there are others who got infected with HIV and STIs. So, it was really tough*.” (Interview with client, male, 24 years) “*Menstrual pads that were once cheap in the shops are now expensive and a lot of girls cannot afford them now. The girls are now becoming fully dependent on CHIEDZA services and making sure that they go there to get the menstrual health products since they are being given for free*.” (Interview with client, female, 24 years)
Provision of ART: longer-term supply through CHIEDZA or through health facilities	“*For those on ART, before we went home on Monday, I think they were given 3 months’ supply so they are safe and I hope the nurses were able to contact everyone and give them their supplies*.” (Interview with youth worker) “*Those we couldn’t reach were forced to go and get their own medication, but I’m sure most of them will not be able to access*.” (Interview with counsellor) “*For those new clients (starting on ART), there is no one who is going to really push them and guide them along that route so probably it might even cause defaulting before they have even started on ART therapy*.” (Interview with community health worker)
Missing social engagement at CHIEDZA	“*If I am stressed, the level of stress would decrease, just by coming to CHIEDZA. During this lockdown I felt worried and anxious. I thought if CHIEDZA had been open, maybe I would have felt fine*.” (Interview with client, female, 24 years)
**Reopening of CHIEDZA as an adapted intervention**	
Reopening of CHIEDZA celebrated	“*Very happy with the reopening of our activities because I believe that the services that we are offering are essential for the adolescents that we are dealing with*.” (Interview with nurse) “*The reopening of CHIEDZA is a great thing because us young people really depend on it*.” (Interview with client, male, 21 years)
Adaptations to CHIEDZA	“*Moving services outside, spacing health booth tents apart, opening one wall for ventilation, wearing masks, wiping table surfaces after use and offering hand washing and sanitiser facilities*” (Non-participant observations of CHIEDZA sites) “*Clients are given a COVID-19-related information session upon arrival while waiting under the tree. The information also included describing and explaining the changes that are being made in COVID-related changes that are being made in CHIEDZA. Clients are receptive to the information being provided*.” (Non-participant observations of CHIEDZA sites) “*Because there are no social activities, the clients are seated and waiting to be served about 25 metres away from where the health booths are (under a tree). They have to sit about 1–2 metres apart, wearing masks. When the hall area has cleared, 3 clients are called at a time to be screened by the youth worker, and the first thing the youth worker is doing is to offer hand sanitizers to the client; and then they are screened for eligibility. Eligible clients are then directed to the relevant health booth (the booths are now situated along one side of the hall with one tent wall open for ventilation). Table surfaces are wiped down with alcohol after every client leaves the health booth*.” (Non-participant observations of CHIEDZA sites)
Response to adaptations	“*Understood the situation, and that it is aimed at safekeeping everyone*” (Interview with client, female, 24 years) “*Maintaining physical distancing is hard to do consistently and at all times, especially for the clients waiting in line to be served*.” (Non-participant observations of CHIEDZA sites). “*I still feel fearful that I might contract the virus*” (Interview with community health worker)
**CHIEDZA was framed as an essential service**	
Authorities questioning what services were essential	“*So ever since we started CHIEDZA besides the COVID lockdown it means I have to go to the police every month to reapply for permissions and that is what we do here. So, (one time when) I went to the police and when I got there, there were so many demands … He was saying would you consider menstruation pads as essential services? Because we are having tonnes and tonnes of young girls leaving their houses and coming to the CHIEDZA centres to get period pain pills and those are not essential services like what you said. And then I said but menstrual hygiene products are part of the essential services of a girl child and they said that this is COVID and we can’t let girls leave their homes to come to CHIEDZA saying they are going there for period pain pills. It’s different from ART, where we are saying that you guys are the ones who are providing the ART care. So, as we have been going along, it’s been having to mix and tuck some of the things that we have been doing to suit the environment that we are working in*.” (Interview with community health worker)
Impact on acceptability of CHIEDZA intervention	“*If young people get to know that it’s all about HIV and STI testing at CHIEDZA, they may avoid attendance: STI services must be provided alongside other services*.” (Interview with client, female, 24 years)
**Increased tension between quantity of clients and quality of service**	
Value placed on time with non-judgemental providers	“*CHIEDZA providers are very free, open and friendly, such that you would continue coming back again, and when you do come back, you won’t fear anything*” (Interview with client, male, 21 years) “*Here I am able to ask any questions because the staff are very friendly, and also I see most of my age mates here, and no one judges any one as compared to the clinic*.” (Interview with client, female, 19 years)
Negative impact of time pressures on quality of service	“*I think that’s the negative impact of COVID. When you are trying to limit the interactions with clients because of the risks of COVID, but at the same time clients need more time to express themselves and what they really need to access from the CHIEDZA providers*.” (Interview with nurse) “*Because of the short time that we have with clients we are now offering a drive-through service, where you come in get what you came for and go. We no longer have a long time with each client to discuss at length and to get to know them better because of the numbers that we have to serve at the short time that is there … As a result, the quality of the service that we offer gets seriously compromised*.” (Interview with nurse) “*Because we are working under conditions where we don’t want to have clients congesting and coming together in large groups, I will be honest and say that the clients are no longer getting as much time as they used to before, like the quality of the service that we used to provide when we were relaxed. There is no time to laugh and talk about general stuff, it’s pressure everywhere, left, right and centre … It makes you start feeling like I am not doing enough, and it will end up making you unfriendly and putting pressure on the clients as well … The pressure is making us divert from being a youth-friendly service*” (Interview with community health worker)
**Impact of adaptations vary by gender**	
Impact of removal of social activities	“*A lot of people are less interested about coming here because others mainly came because of the entertainment. Now that it is not there, they are no longer coming as before*.” (Interview with client, male, 19 years) “*It feels like a party without the music: it’s no longer fun like it used to be.*” (Interview with community health worker) “*We used to enjoy playing pool but because it is no longer there it is a bit boring but I am glad we can still get the services.*” (Interview with client, female, 19 years)
Particular impact for young men	“*And then sometimes for boys especially before lockdown, most of them used to come because of the games and music that were entertaining, of which the situation has changed now.*” (Interview with client, female, 24 years) “*Boys are the ones who are pleading that they shouldn’t come to the centres for condoms only.*” (Interview with client, male, 21 years) “*There really is a challenge in attracting male clients*” (Interview with youth worker)
Intervention response to mobilise more young men	“*What we do is that we just explain them that we will take men first, because they come in few numbers … and the consult of the young men are usually shorter … So, she will not feel the impact a lot that someone has skipped the line.*” (Interview with community health worker) “*At our sites boys come and they form a separate queue because honestly speaking we want them and our numbers are low with regards to guys so we have to prioritise them. The girls would start complaining saying, hey, we cook at home but you don’t think about that, we would have excused ourselves from home, and then the boys just come and do as they please here.*” (Interview with community health worker)

### Sudden closure: disrupted access to HIV and SRH services for youth

The sudden closure of CHIEDZA sites severely impeded youth’s access to SRH services. This was accompanied by the closure of almost all youth health services due to the government-mandated lockdown. This led to reduced access to family planning and condoms, with many clients reporting that they were “*engaging in unprotected sex*” (interview with client, female, 24 years). Youth living with HIV were prioritised and were given three-months’ supply of anti-retroviral treatment (ART) when CHIEDZA closed. Those who needed to be linked to additional care were followed up individually. Those who were not reached had to go to their local health facility to collect ART, often with travel and cost implications. Some young women, unable to access menstrual hygiene products from CHIEDZA, were forced to use and wash pieces of cloth instead. SRH needs intersected with other needs, including reported increases in intimate partner violence (“*you can hear people fighting in their homes*”), economic insecurity, food insecurity, and social isolation. Clients discussed the psychosocial impacts of the sudden closure, including missing feeling “*boosted*” and “*uplifted*” by the security offered by CHIEDZA; a feeling exacerbated by needing such support even more during this acutely stressful time and social isolation.

### Reopening of CHIEDZA as an adapted intervention

When CHIEDZA was allowed to reopen, both providers and clients were happy and agreed that “*the reopening of CHIEDZA is a great thing because us young people really depend on it*” (interview with client, male, 21 years). However, in order to reopen, it was necessary to implement major and rapid adaptations to comply with national guidance and COVID-19 prevention measures. Although as much as possible of the core intervention was maintained, these adaptations impacted access to service provision, as well as acceptability and engagement with the intervention.

### Justifying CHIEDZA’s status as an essential service

For CHIEDZA to continue to operate while COVID-19 restrictions were in place, the programme and provider teams had to continually demonstrate to the authorities compliance with all measures and the essential role the intervention served. What constituted “essential services” was determined by council, police and government authorities. While ART was unequivocally deemed “essential”, authorities considered that the provision of menstruation pads, analgesia for period pain, condoms, and contraceptives (particularly long-acting reversible contraceptives) were not essential services. CHIEDZA providers had to vehemently argue to authorities that the latter services were also necessary components of this youth service. Some clients were stopped by police on their way to CHIEDZA and asked to justify where they were going and why. Clients who had previously justified their attendance to others by emphasising their need for menstrual hygiene products, for example, even though they may also be accessing HIV care or STI testing during the same visit, had to instead emphasise the latter when stopped by police. This risked reframing the branding of CHIEDZA and as one client said, “*if young people get to know that it’s all about HIV and STI testing at CHIEDZA, they may avoid attendance*” (interview with client, female, 24 years). Where possible, CHIEDZA continued to frame the service to clients, communities, and even the authorities, as integrated and for all youth, in order to preserve the social acceptability of attending the intervention.

### Increased tension between quantity of clients and quality of service

Being able to have time with high-quality, non-judgemental healthcare providers is a core pillar of the CHIEDZA intervention, and was described by clients as a key reason that they regularly attended CHIEDZA: “*CHIEDZA providers are very free, open and friendly, such that you would continue coming back again*” (interview with client, male, 21 years). However, the changed opening hours (due to government restrictions that all businesses must close at 3.30pm), and restrictions in the number of clients at the intervention site at any one time to maintain physical distancing requirements (due to the providers feeling pressure to see waiting clients quickly), effectively reduced the time providers could spend with each client. For providers, these time pressures made it more difficult to give sufficient time to talk with the clients and to support them to open up, meaning that “*the quality of the service that we offer gets seriously compromised*” (interview with nurse). Providers felt increased stress and that “*the pressure is making us divert from being a youth-friendly service*” (interview with community health worker). Acknowledging provider and client concerns, further adjusting timings where feasible in line with government regulations, and supporting providers to encourage their continued motivation, were measures taken to attempt to reduce the negative impact.

### Impact of adaptations vary by gender

Social activities had previously been an important attraction for youth to come to CHIEDZA, particularly for young men. While the need to remove social activities for infection prevention was understood, providers and clients alike described CHIEDZA as having become a less attractive place to come and spend time: “*it feels like a party without the music: it’s no longer fun like it used to be*” (interview with community health worker). Women continued to attend to access much needed services. However, the removal of social activities particularly impacted young men’s attendance, who reported that condoms were not a sufficient attraction to attend. This led to adaptations to the intervention’s mobilisation strategy: “*we have had to re-strategize so that we get male clients*” (interview with youth worker). Adaptations included enabling young men to skip the queue for services and prioritising males for free transport from the community to the intervention site. However, this strategy provoked resentment among some young women. It was reported to be difficult to strike the delicate line between encouraging male attendance, while not reproducing gender inequalities by prioritising men above women.

## Discussion

SRH youth services were challenged by having to operate within the restrictions imposed by the response to COVID-19. During the closure of CHIEDZA, SRH needs continued, but were unmet, and were seen to be embedded in broader psycho-social and health needs, in the context of COVID-19. We provide a case study of re-establishing the provision of a community-based youth-centred service during COVID-19, through rapid and continuous adaptation of services, that aimed to mitigate the documented collateral damage of fractured access to SRH services.^3,16,17^ The results demonstrate the ramifications the adaptations have on service provision, access and acceptability. Our examination of the removal of key aspects of a youth-friendly intervention highlights lessons about what is fundamental in the provision of youth SRH services.

Continued service provision during the COVID-19 pandemic is critical to prevent corroding hard-won gains in SRH. We share lessons about creative approaches to reducing COVID-19 risk, while supporting the continuation of service delivery which can, in turn, reduce the threat posed to youth SRH outcomes. Our study illustrates that certain adaptations are viable for the medium term, albeit with consequences on the delivery and acceptability of the intervention, thereby highlighting a tension between viability and acceptability of intervention adaptations. Minimising crowding, mandating mask-wearing, providing personal protective equipment for health providers and hand-washing facilities, moving services outdoors, were all feasible adaptations to youth SRH services, in line with WHO recommendations for delivering health services to mitigate the risk of COVID-19 transmission.^18^ The subsequent addition of a mobile-based information text service, which coincided with the COVID-19 pandemic, had a high uptake and supports the success of other programmes which adapted to online delivery.^19^ However, the physical service provision was considered essential, both for delivery of commodities and testing, and for face-to-face psychosocial support, and aligns with other studies which show the need for in-person spaces and support for youth.^20^

However, concurrently, some of the adaptations undermined some core aspects of the intervention. The added pressure on providers and reduced time with clients, alongside documented work-related burden and strain that such infection prevention and control adaptations put on healthcare providers,^21^ may have serious ramifications on the “youth-friendly” qualities of the intervention. Government authorities’ prioritisation of services into essential and non-essential services did not align with youth clients’ own prioritisation, and exposed clients’ needs for services that are less socially acceptable, with an impact on access and acceptability. There are, therefore, tensions between physical safety (in terms of risk of COVID-19 transmission) and social safety for youth (in terms of judgment related to exposed access to SRH services), with the balance needed so that focus on reducing physical risk does not eclipse the social risk. The danger is that this may translate into more substantial physical risks for youth if they are unable to seek adequate prevention or treatment for SRH; physical risks which may be greater to them than the direct health risks of COVID-19. Our study has highlighted that we continue to primarily respond to SRH needs through a clinical focus on physical health. The experiences of CHIEDZA clients during the COVID-19 pandemic illuminates that meaningful SRH protection requires interventions serving a much wider remit than physical health, by incorporating a focus on the social, structural, and economic determinants, which shape sexual risk, mental health, and acceptability of SRH services.

Through necessarily having to adapt components of the intervention, including the social activities, the physical set-up of the intervention, and the time with clients, it has clarified broader learning about what is fundamental about provision of youth SRH services. The ramifications that the removal of social activities had on youth access, engagement, and uptake of services demonstrate the integral value of social activities. It demonstrates that entertainment and social activities, which are not currently prioritised,^22^ are not an optional extra should funding allow, but rather key components of a youth-friendly package, to add to the toolbox of strategies to tackle the challenge of engaging young men.^23^ The impact of authorities’ framing CHIEDZA as an essential service on acceptability of the intervention reiterates the importance of integrated service delivery to enable youth to access a range of services while not exposing what service they require.^24^ It further demonstrates the importance of community participation, in this case with youth, in devising how to adapt services which mitigate risk but continue to meet their needs in order to minimise disruption to services and health outcomes, even in a time of crisis.^25^ It has been widely recognised that there has been inadequate community engagement in the response to COVID-19, particularly at the start of the pandemic.^26,27^ Engagement with communities in guiding intervention decisions is even more critical during times of flux and crisis, but in reality, it happens infrequently.^28^ The concerns around the compromised quality of service provision highlight that high-quality youth-friendly service provision not only takes provider training^29^ but requires reducing provider pressure, supporting provider motivation, and, importantly, providing sufficient time.

## Conclusions

We have highlighted some of the key qualities of youth HIV and SRH services which provide important lessons both for the design of future youth-friendly interventions, but also for thinking about how to navigate the gaps and challenges that adaptations to COVID-19 prevention measures expose. Many of the consequences of adapted interventions are not insurmountable. Programmes must be increasingly flexible to enable adaptations based on learnings from the efficacy and consequences of adaptations to COVID-19 and to continue to enable access, albeit adapted, to SRH services for youth.
